# Transferrin-conjugated doxorubicin-loaded lipid-coated nanoparticles for the targeting and therapy of lung cancer

**DOI:** 10.3892/ol.2014.2840

**Published:** 2014-12-30

**Authors:** YAJUN GUO, LIJUAN WANG, PENG LV, PENG ZHANG

**Affiliations:** 1 Department of Nursing, The Fifth Affiliated Hospital of Zhengzhou University, Zhengzhou, Henan 450052, P.R. China; 2 Department of Cardiothoracic Surgery, Tianjin Medical University General Hospital, Heping, Tianjin 300052, P.R. China

**Keywords:** transferrin, tumor targeting, lung cancer, doxorubicin

## Abstract

In the present study, a targetable vector was developed for the targeted delivery of anticancer agents, consisting of lipid-coated poly D,L-lactic-*co*-glycolic acid nanoparticles (PLGA-NP) that were modified with transferrin (TF). Doxorubicin (DOX) was used as a model drug for lung cancer therapy. The use of these NPs combined the advantages and avoided the disadvantages exhibited individually by liposomes and polymeric NPs during drug delivery. The lipid coating of the polymeric core was confirmed by transmission electron microscopy. The physicochemical characteristics of transferrin-conjugated lipid-coated NPs (TF-LP), including the particle size, zeta potential, morphology, encapsulation efficiency and *in vitro* DOX release, were also evaluated. The cellular uptake investigation in the present study found that TF-LP was more efficiently endocytosed by the A549 cells, than LP and PLGA-NPs. Furthermore, the anti-proliferative effect exhibited by DOX-loaded TF-LPs on A549 cells and the inhibition of tumor spheroid growth was stronger compared with the effect of DOX-loaded lipid-coated PLGA-NPs and PLGA-NPs. In the *in vivo* component of the present study, TF-LP demonstrated the best inhibitory effect on tumor growth in the A549 tumor-bearing mice. It was concluded that TF-LP may be an efficient targeted drug-delivery system for lung cancer therapy.

## Introduction

Lung cancer is characterized by uncontrolled cell growth in lung tissues, which results in metastasis, the invasion of tissues adjacent to the lesion and infiltration beyond the lungs. In 2010, lung cancer accounted for >0.15 million deaths in the USA, and >0.2 million cases are registered annually (1). Despite surgery being the preferred method for the removal of cancer, it cannot completely excise the affected tissue and supplementary multi-drug chemotherapy or radiation may be required. The current drugs of choice for lung cancer therapy include etoposide, docetaxel, doxorubicin (DOX), carboplatin and cisplatin (2). However, limited therapeutic action has been demonstrated by the preferred chemotherapeutic agents for cancer therapy.

Numerous nanoparticle (NP)-based therapies have been approved for clinical used or have entered clinical development over the previous two decades (3). Liposomal drugs (4) and polymer-drug conjugates (5) are two leading classes of NP-based therapy and account for the majority of the products approved for clinical use. Liposomes and polymer-NPs each possess advantages and disadvantages. Polymeric NPs exhibit an elevated loading capacity for hydrophobic drugs compared with liposomes and drug release is generally dominated by polymer degradation and drug diffusion in polymeric NPs, which can be controlled through the use of proper polymers that exhibit a desirable degradation rate and binding affinity with the encapsulated drugs (6,7). Advantages of liposomal formulations include the ability to carry hydrophilic and hydrophobic drugs within the aqueous vesicles and lipid bilayer membranes, respectively. The liposomal formulations also exhibit a high biocompatibility, providing protection for the drugs from the external environment, and easily undergo surface modification with other molecules, including polyethylene glycol (PEG), and targeting ligands, which achieves an improved systemic circulation lifetime and targeted drug delivery, respectively (8,9). However, these formulations also possess a short shelf life due to the preparation and purification of liposomes involving relatively complicated steps, the low loading efficiency for hydrophobic drugs, the burst-release kinetics of encapsulated drugs and the instability of the formulation during storage.

In the present study, lipid-coated poly D,L-lactic-*co*-glycolic acid (PLGA) NPs (L-P) were prepared, which combined the respective benefits of liposomes and polymer-NPs and avoided their respective disadvantages. The lipid-coated NPs comprised a biodegradable and biocompatible hydrophobic polymeric core that was comprised of PGLA, a monolayer of phospholipids and an outer corona layer of PEG. The biocompatibility, biodegradability and sustained drug-release of these NPs, as well as the easy surface modification with other molecules that include PEG and targeting ligands, which achieves a prolonged systemic circulation lifetime and targeted drug delivery, the excellent stability in the blood, and, crucially, the high drug-loading yield, makes L-Ps a promising drug delivery system (10). These properties provide the basis for a stable, high-payload targeted drug delivery vehicle that possesses the potential to maximize the chemotherapeutic efficacy of anti-cancer agents on the target cancer cells.

For the targeting ligand, transferrin (TF) was selected as a basis since the TF receptor is overexpressed in 90% of tumors (11,12). In the present study, TF-conjugated lipid-coated NPs (TF-LPs) were successfully prepared and characterized. The DOX-loaded TF-LPs (TF-LP-DOX) demonstrated elevated cytotoxicity against lung cancer cells and an improved therapeutic effect in the lung cancer-bearing nude mice compared with their non-targeted counterparts.

## Materials and methods

Ester-terminated PLGA, with a 50:50 monomer ratio and a viscosity of 0.50–0.85 dl/g, was purchased from Shandong Key Laboratory of Medical Polymeric Material (Jinan, Shandong, China). Soybean lecithin, comprising 90–95% phosphatidylcholine and mPEG_2000_-DSPE and Mal-PEG_2000_-DSPE, was purchased from Avanti Polar Lipids, Inc. (Alabaster, AL, USA). TF was obtained from Sigma-Aldrich (St. Louis, MO, USA). DOX was purchased from Zhejiang Haizheng Pharmaceutical Co., Ltd. (Taizhou, Zheijiang, China). Other chemicals and reagents were of analytical grade and obtained commercially.

BALB/c male athymic nude mice, ~20 g in weight, were purchased from the Experimental Animal Center of Tianjin Medical University (Heping, Tianjin, China). All animal experiments adhered to the principles of care and use of laboratory animals and were approved by the Experimental Animal Administrative Committee of Tianjin Medical University. This study was approved by the ethics committee of Zhengzhou University (Zhengzhou, China).

### The preparation of PLGA-NPs

DOX-loaded PGLA-NPs (PGLA-NP-DOX) were prepared using the water in oil in water double emulsion method (13,14). Briefly, 20 mg of mPEG-PLGA was dissolved in 1 ml of methylene chloride. Water or DOX solution (0.2 ml) was then transferred to a centrifuge tube, and the mixture was emulsified by sonication for 3 min. The emulsion and 2 ml of 2% polyvinyl alcohol (PVA) were then emulsified by sonication for 5 min. Subsequently, the emulsion was slowly dropped into 10 ml of 0.6% PVA and stirred for 10 min at room temperature. Following vacuum evaporation of the solvent, the NPs were collected by centrifugation at 18,000 × g for 10 min at room temperature and were washed twice using distilled water.

### Preparation of the L-Ps and TF-LPs

The DOX-loaded L-Ps (LP-DOX) were prepared as previously described (15,16). Briefly, PLGA was initially dissolved in acetone, and lecithin and mPEG-DSPE2000 (15% of the PLGA polymer weight; mole ratio lecithin:mPEG-DSPE2000, 7.5:2.5) were dissolved in a 4% ethanol aqueous solution and heated to 65°C. The PLGA acetone solution was then added into the preheated lipid aqueous solution drop-wise (1 ml/min) under gentle stirring, which was followed by vortexing for 3 min. The NPs were left for 2 h to self-assemble, with continuous stirring, until the organic solvent was evaporated. The remaining organic solvents were removed under reduced pressure at 37°C. The final concentration of PLGA in NP suspensions was set to 1 mg/ml with distilled water. The NPs were used immediately, stored at 4°C, or freeze-dried in liquid nitrogen and lyophilized for storage at −80°C for later use.

The TF-LP-DOX were prepared using the post-insertion method (17,18). Firstly, the TF was reacted with Traut’s reagent at a molar ratio of 1:5 to yield TF-SH. Secondly, the TF-SH was reacted with the micelles of DSPE-PEG_2000_-Mal at a molar ratio of 1:10, and then incubated with L-P for 1 h at 37°C. The ratio of TF-PEG_2000_-DSPE to lipid was 1:50. The final particles were stored at 4°C for further experiments.

### Characterization of the NPs

#### Size and zeta-potential measurements

The size and zeta potential of the NPs were measured using a dynamic light scattering detector (Zetasizer Nano-ZS90; Malvern Instruments, Worcestershire, UK).

#### Drug encapsulation efficiency (EE) and drug loading coefficient

The free DOX was removed by passing through a Sephadex G-50 column. The quantity of DOX encapsulated in the NPs was measured by high performance liquid chromatography (HPLC; Agilent LC1200; Agilent, Santa Clara, CA, USA). A reversed phase Inertsil®ODS-3 column (150–4.6 mm; pore size, 5 mm; GL Sciences Inc., Shinjuku, Japan) was used. Freeze-dried NPs (3 mg) were dissolved in 1 ml DCM. Subsequent to the evaporation of DCM, 3 ml mobile phase (50:50 v/v acetonitrile/water solutions) was added to dissolve the drugs. The solution was then filtered by a 0.45 mm polyvinylidene fluoride syringe filter for HPLC analysis. The column effluent was detected at 227 nm using an ultraviolet/visible detector. The EE and drug loading content were calculated as follows: EE (%) = (amount of drug encapsulated in NPs/initial amount of drug used in the fabrication of NPs) × 100; and drug loading content (%) = (amount of drug encapsulated in NPs/amount of drug encapsulated in NPs and excipients added) × 100.

#### Stability of NPs

To demonstrate the serum stability of lipid-coated NPs, the particle sizes and turbidity variations of the NPs were monitored in the presence of fetal bovine serum (FBS) (19,20). Briefly, the NPs were mixed with an equal volume of FBS at 37°C by gentle agitation at 36 × g. At the predetermined time-points of 1, 2, 4, 8 and 24 h, 200 μl of the sample was pipetted onto a 96-well plate and the transmittance was measured at 750 nm using a microplate reader (Varioskan Flash; Thermo Fisher Scientific, Waltham, MA, USA). Another 200 μl was diluted to 1 ml using 5% glucose solution for the particle size measurements obtained by the Zetasizer Nano ZS90 light scattering detector (Malvern Instruments).

#### In vitro drug release

The release kinetics of DOX from DOX-loaded PLGA-NP, LP and TF-LP in phosphate-buffered saline (PBS) were evaluated using a dialysis method for ≤4 days. The samples were individually dispersed in 5 ml of the PBS and were placed into a cellulose membrane dialysis tube (MW cut off, 12,000–14,000). The dialysis tube was then placed into 195 ml of PBS and the release test was performed at 37°C with a centrifugation rate of 320 × g. At predetermined time points, 1 ml release medium was taken, refilled with the same amount of the fresh medium, and concentrations of the released drug were determined by RP-HPLC, as aforementioned.

### In vitro cellular uptake

A549 cells were grown in RPMI-1640 medium (HyClone, Logan, UT, USA) that contained 10% FBS, 100 μg/ml of streptomycin and 100 units/ml of penicillin. The cells were maintained at 37°C in a humidified incubator with 5% CO_2_.

For the quantitative study, the A549 cells were harvested with 0.125% trypsin-EDTA solution (Invitrogen, Carlsbad, CA, USA) and seeded into 24-well assay plates (Corning Inc., Corning, New York, NY, USA) at 10^5^ viable cells/well. Subsequent to the cells reaching confluence, the cells were incubated with 100 μl of 10 μg/ml DOX-loaded NPs, all three types, in the 1640-medium supplemented with 10% HyClone FBS (Thermo Scientific) and 1% penicillin-streptomycin (Invitrogen) at 37°C for 2 or 4 h. At the designated time period, the suspension was removed and the wells were washed three times with 1,000 μl cold PBS. Subsequently, 50 μl of 0.5% Triton X-100 was introduced into each well for cell lysis. The fluorescence intensity of each sample well was measured by a microplate reader (GENios; Tecan, Männedorf, Switzerland) with an excitation wavelength of 480 nm and an emission wavelength of 580 nm.

For the qualitative study, A549 cells were harvested using 0.125% trypsin-EDTA solution (Invitrogen) and seeded in LABTEK cover glass chambers (Nalge Nunc International, Rochester, NY, USA) having RPMI-1640 at a concentration of 5×10^3^ viable cells/chamber. The cells were incubated overnight and were subsequently incubated with DOX loaded NPs in the RPMI-1640 (concentration of 10 μg/ml) at 37°C. After 4 h, the cells were washed 3 times with cold PBS and fixed by 4% paraformaldehyde for 20 min. Then, the cells were washed twice with cold PBS. The nuclei were stained by incubating the cells with DAPI (Roche Diagnostics, Basel, Switzerland) for an additional 10 min. The cell monolayer was washed three times with PBS and observed by confocal laser scanning microscopy (CLSM; Leica, Germany).

### In vitro cytotoxicity and anti-proliferation assay

Comparison between the *in vitro* cytotoxicity and tumor cell proliferation of A549 cells in response to various formulations was performed using the sulforhodamine B (SRB) colorimetric assay. In brief, 4,000 A549 cells were seeded into 96-well plates and incubated overnight. The cells were then exposed to serial concentrations of various DOX formulations in the culture medium for 48 h at 37°C. Subsequently, the cells were fixed with trichloroacetic acid, washed and stained by SRB. The absorbance was measured at 540 nm using a 96-well plate reader (Bio-Rad Laboratories, Hercules, CA, USA). Dose-response curves were generated, and the concentration of drug that resulted in 50% cell death (IC_50_) was calculated using Origin 7.0 software (OriginLab, Northampton, MA, USA).

### Evaluation of tumor spheroid penetration

To prepare the three-dimensional tumor spheroids, A549 cells were seeded at a density of 2×10^3^ cells/200 μl per well in 96-well plates coated with 80 μl of a 2% low-melting-temperature agarose. Seven days after the cells were seeded, the tumor spheroids were treated with 10 μg/ml DOX-loaded NPs. After 4 h of incubation, the spheroids were rinsed three times with ice-cold PBS and fixed with 4% paraformaldehyde for 30 min. The spheroids were then transferred to glass slides and covered by glycerophosphate. The fluorescent intensity was observed by laser scanning confocal microscopy (Leica Microsystems GmbH, Wetzlar, Germany).

### Growth inhibition of tumor spheroid

The tumor spheroids were prepared as aforementioned for the evaluation of tumor spheroid penetration. Seven days later, the spheroid-containing wells were treated with 0.8 mg/ml of DOX solution, and DOX-loaded NPs. The length and width of each spheroid was measured every day for eight days and the volume was calculated. A volume curve was drawn to compare the effect of each treatment with the various formulations.

### In vivo imaging

The DIR-loaded NPs were utilized as previously described to investigate the distribution of NPs in lung cancer A549 cell-bearing nude mice. The nude mouse lung cancer xenograft model was established by subcutaneously injecting A549 cells (1×10^7^ cells per animal) into the backs of 4–6 week-old BALB/c male athymic nude mice. The DiR-loaded NPs were injected into A549 lung cancer-bearing nude mice via intravenous administration, and then the *in vivo* fluorescence imaging was performed using the IVIS Spectrum system (Caliper Life Sciences, Hopkinton, MA, USA).

### Statistical analysis

Analysis of variance was used to assess the variance of the whole values in each group. Statistical significance was evaluated using Student’s t-test for the comparison between experimental groups. P<0.05 was considered to indicate a statistically signficant difference.

## Results and Discussion

### Characterization of the NPs

#### Particle size, size distribution, drug encapsulation efficiency and drug-loading efficiency

**Figure 1:**
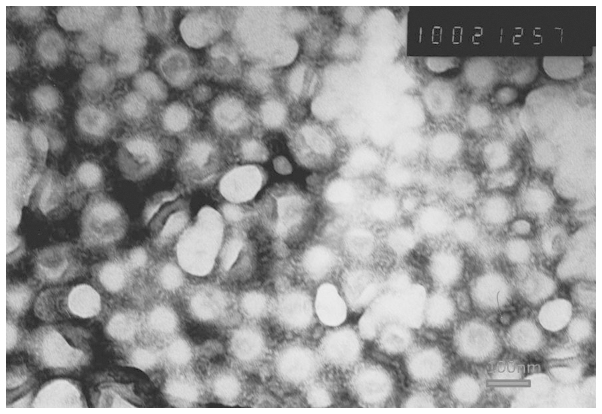
Transmission electron microscopy image demonstrating the lipid-coated structure of the NPs. The NPs were negatively stained with uranyl acetate to enhance the electron contrast between the polymers and the lipids. N-Ps, nanoparticles.

Transmission electron microscopy was used to observe the shape and surface morphology of the investigated NPs (Fig. 1). The NPs were all revealed by microscopy to exhibit a uniform spherical appearance that indicated the successful formation of the lipid-coated NPs. The conventional DOX-loaded NPs were, on average, ~110 nm in diameter, with a PDI of 0.200 (Table I). In order to justify the clinical application of NPs, the drug encapsulation efficiency (EE) is crucial. The EE of the three types of NPs formulations are reported in Table I. These EE values are reasonable and confirm the effectiveness of lipid-coated NPs for loading anticancer drugs. Evidently, the present formulation system reveals the potential for a useful and practical drug delivery carrier with an appropriate size, stability and drug loading capacity.

#### Stability of DOX-loaded NPs

**Figure 2:**
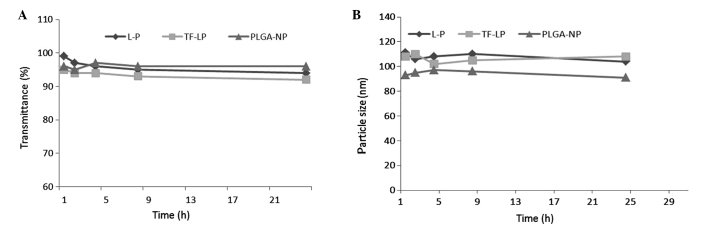
(A) The variation in transmittancy versus the various incubation times of nanoparticles at the wavelength of 750 nm when incubated with phosphate-buffered saline containing 50% (v/v) FBS for 24 h at 37°C (n=3). (B) The variation in particle sizes of nanoparticles in 50% FBS. FBS, fetal bovine serum; PLGA-NP, poly D,L-lactic-co-glycolic acid nanoparticles; L-P, lipid-coated PGLA-NPs; TF-LP, transferrin-modified LPs.

As particle stability in physiological conditions is a prerequisite for the further application of NPs *in vivo*, 50% FBS was employed to mimic the *in vivo* conditions. Particle sizes and transmittance variations as important parameters were monitored in the present study to explore the serum stability of NPs. As reported in Fig. 2, the particle sizes and transmittance have hardly changed for L-P and TF-LP over 24 h, indicating that there was no aggregation in the presence of serum.

#### In vitro drug release

**Figure 3:**
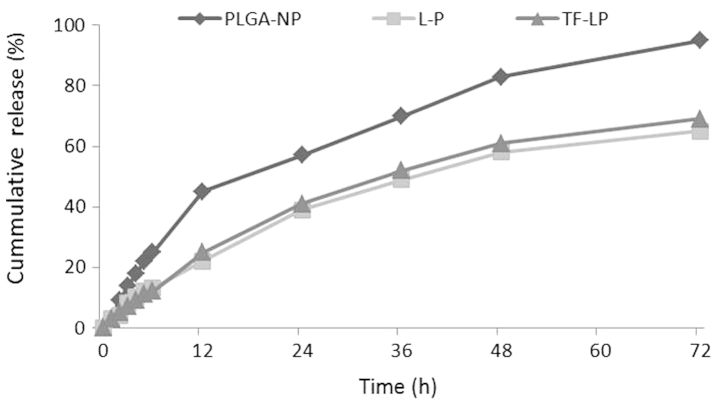
*In vitro* doxorubicin release profile from PLGA-NPs, L-Ps and TF-LPs. Phosphate buffered saline (0.1 M; pH 7.4) was selected as the release medium. The nanoparticle dispersion was agitated in an orbital shaker at 160 × g, in a water bath at 37°C. high performance liquid chromatography was performed to measure the released drug concentration (n=3). PLGA-NP, poly D,L-lactic-co-glycolic acid nanoparticles; L-P, lipid-coated PGLA-NPs; TF-LP, transferrin-modified L-Ps.

The present study investigated the release of DOX *in vitro* from PLGA-NP, L-P and TF-LP. The release profile of these three groups is shown in Fig. 3. DOX was released at a higher rate from PLGA-NPs compared with the other groups. As shown in Fig. 3, PLGA-NPs demonstrated almost 95% drug release within three days. Conversely, L-Ps and TF-LPs produced only ~65% leakage within three days.

### Cellular uptake

**Figure 4:**
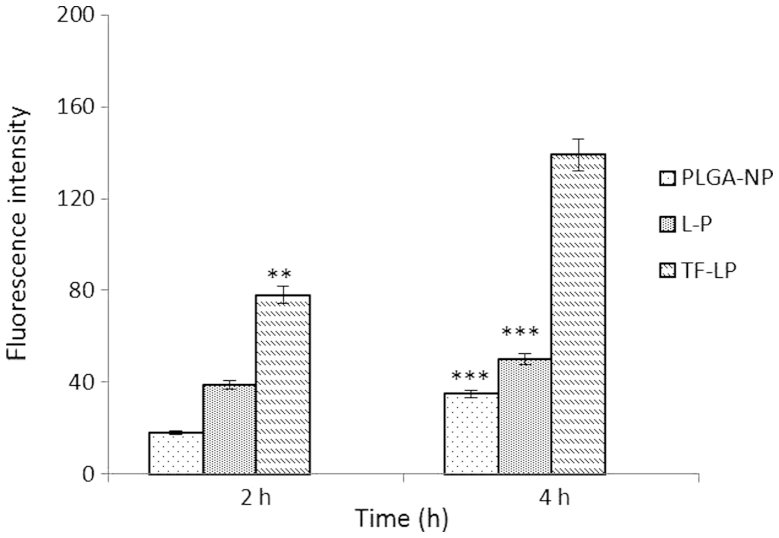
Measurement of *in vitro* uptake of doxorubicin-loaded PLGA-NPs, L-Ps and TF-LPs by A549 cells. Data represented the mean ± standard deviation (n=3). Compared with TF-LP at 4 h, ^**^P<0.01 and ^***^P<0.001. PLGA-NP, poly D,L-lactic-co-glycolic acid nanoparticles; L-P, lipid-coated PGLA-NPs; TF-LP, transferrin-modified LPs.

**Figure 5:**
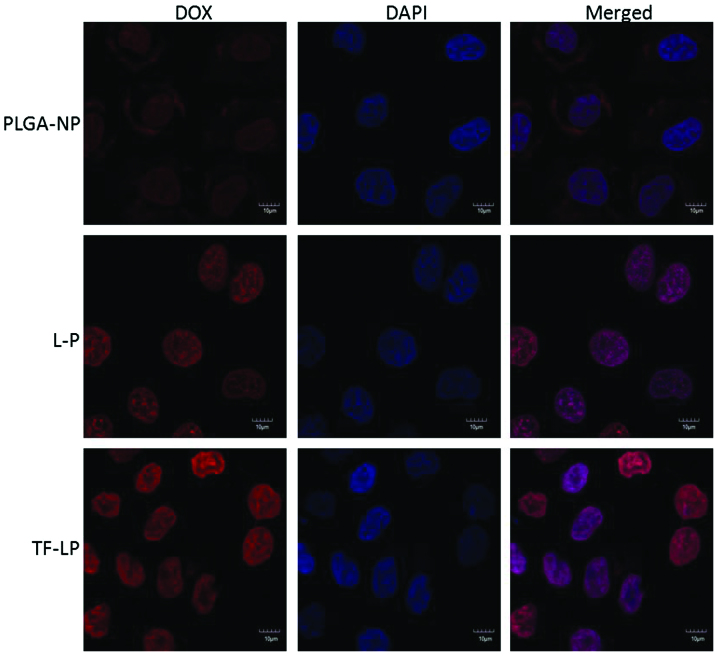
Confocal laser scanning microscopy images demonstrating the internalization of fluorescent nanoparticles in cells following a 4-h incubation. In the DOX column, the red fluorescence demonstrates DOX-loaded nanoparticles distributed in cytoplasm. In the DAPI column, the DAPI channels exhibt blue fluorescence from DAPI-stained nuclei. In the merged column, the merged channels of DOX and DAPI channels are shown. Scale bar, 10 μm. DOX, doxorubicin; PLGA-NP, poly D,L-lactic-co-glycolic acid nanoparticles; L-P, lipid-coated PGLA-NPs; TF-LP, transferrin-modified LPs.

The A549 cells were able to take up the DOX-loaded PLGA-NP, L-P and TF-LP at various capacities (Fig. 4). The TF-LP uptake was ~2.8 and 4.1 times higher compared with L-P and PLGA-NP, respectively. A similar result was obtained for the targeting capacity of TF receptors. The fluorescence intensity of TF-LP in the A549 cells was significantly higher when compared with PLGA-NP and L-P (P<0.001). The quantitative results indicated analogous results to the fluorescence imaging shown in Fig. 5. Due to the existence of lipids analogous to cell membrane components on the surface of L-P, the uptake of the L-P in A549 cells is facilitated by the mutual interaction between L-P and the cell membrane, resulting in an elevated uptake efficiency compared with PLGA-NP. For TF-LP, the receptor-mediated endocytosis (RME) may facilitate the cellular uptake, resulting in an increased uptake efficiency compared with L-P.

### In vitro cytotoxicity and anti-proliferation assay

The cytotoxic effects of the various DOX formulations on A549 cells are summarized in Table II. The efficacy of DOX-loaded NPs was improved by modification with TF. In particular, TF-LP resulted in decreases of 33.8 and 64.8% in the IC_50_ values compared with L-P and PLGA-NPs after 48-h incubation with A549 cells, respectively.

### Evaluation of tumor spheroid penetration

**Figure 6:**
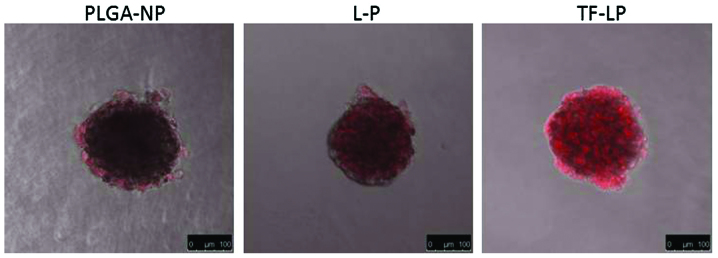
Confocal laser scanning microscopy images showing the uptake of doxorubicin-loaded PLGA-NP, L-P and TF-LP by A549 tumor spheroids at 4 h. Scale bar, 100 μm. PLGA-NP, poly D,L-lactic-co-glycolic acid nanoparticles; L-P, lipid-coated PGLA-NPs; TF-LP, transferrin-modified LPs.

There are hypoxic and avascular regions in numerous solid tumors. As delivery systems exhibit poor permeation, a low quantity of the drug accesses the interior of solid tumors. Tumor spheroids were prepared as they lack blood vessels, which mimics the *in vivo* status of tumors (21–23). The tumor spheroid is an invaluable tool for the evaluation of the solid tumor penetration effect of NPs. Confocal laser scanning microscopy images of 3D tumor spheroids 4 h subsequent to the application of DOX-loaded PLGA-NP, L-P and TF-LP are shown in Fig. 6. The present results indicated that the presence of TF-targeting ligand enhanced solid tumor penetration.

### Growth inhibition of tumor spheroids

**Figure 7:**
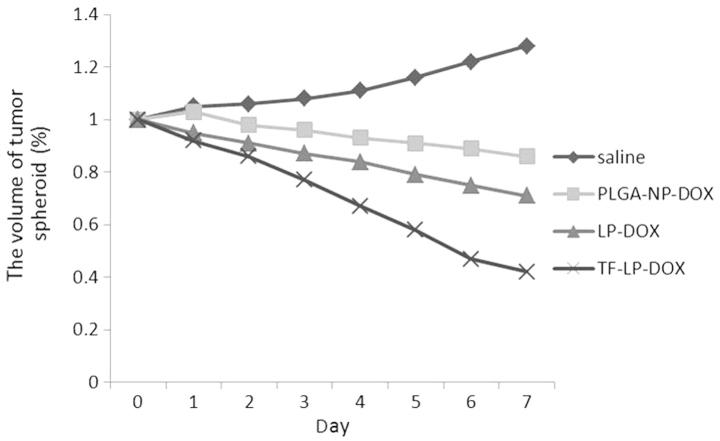
Percentage change in ratios of tumor spheroid volume subsequent to the application of various DOX formulations and in the saline blank control. DOX, doxorubicin; PLGA-NP-DOX, DOX-loaded poly D,L-lactic-co-glycolic acid nanoparticles; LP-DOX, DOX-loaded lipid-coated PGLA-NP; TF-LP-DOX, transferrin-modified DOX-loaded lipid coated NPs.

The present study also investigated the effect of various treatments on the growth of tumor spheroids. The volume ratios of the *in vitro* tumor spheroids subsequent to treatment with saline, PLGA-NPs, L-Ps and TF-LPs at the final DOX concentration of 0.25 mg/ml are shown in Fig. 7. In the absence of any drug, the tumor spheroids were observed to continue to increase in size and volume, reaching 128% of the primary volume after seven days. A marked reduction in the volume of tumor spheroids was observed in all DOX formulations after seven days of treatment, indicating that the tumor spheroids were sensitive to DOX. The percentage change in the ratios of tumor spheroid volumes on day seven was almost 86, 71 and 42% for the PLGA-NP, L-P and TF-LP groups, respectively. The present results indicated that the inhibitory effects of DOX on the 3D tumor spheroids was significantly improved by TF-LP. Solid tumors contain high-pressure regions with few vessels. This *in vivo* status was successfully imitated as the tumor spheroids lacked blood vessels, and the elevated inhibitory effect indicates that TF-LP may improve the *in vivo* therapeutic effect of chemotherapeutic agents.

### In vivo near-infrared (NIR) imaging

**Figure 8:**
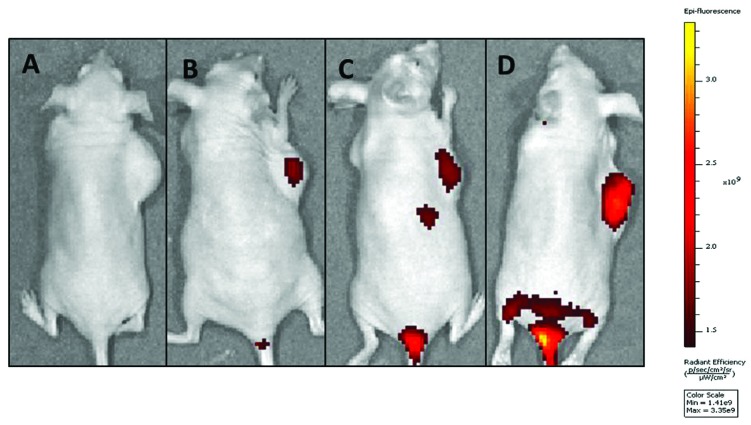
*In vivo* investigation. Image of the mice that were anesthetized 24 h after intravenous injection of various types of 1,1′-Dioctadecyl-3,3,3′,3′-tetramethylindotricarbocyanine iodide-loaded nanoparticles, respectively. Mice were injected with (A) saline, (B) PLGA-NP, (C) L-P or (D) TF-LP. Imaging revealed that the accumulation of nanoparticles in the tumor was highest for TF-LP, when compared with the other nanoparticles. PLGA-NP, lipid-coated poly D,L-lactic-*co*-glycolic acid nanoparticles; L-P, lipid-coated PGLA-NPs; TF-LP, transferrin-modified LPs.

A NIR reflection fluorescence probe 1,1′-Dioctadecyl-3,3,3′,3′-tetramethylindotricarbocyanine iodide (DIR) was encapsulated in each NP to trace the NP delivery behavior in mice. As shown in Fig. 8, the signal intensity in the tumor of TF-LPs at 24 h was stronger compared with the other groups, which indicated an elevated lung cancer-targeting property of TF-LPs. There were NIR reflection fluorescent signals in the excised tumor of each group, and the intensity of the fluorescence in the TF-LP group was the strongest compared with all the other groups, indicating an increase in the delivery of drug to the tumor. Control animals injected with saline solution produced no fluorescent signals, which confirmed that the observed fluorescent signal in the experimental groups was derived from the NPs. It was also observed that there was a slight difference between the fluorescence intensity in the groups treated with L-P and PLGA-NP. The biodistribution experiments (Fig. 8) indicated that TF-LP, as a drug delivery carrier *in vivo*, was also able to specifically target therapeutic agents to tumors that overexpress the TF receptor via TF. It was hypothesized that the high accumulation effect and the strongest fluorescence intensity of the TF-LP group were achieved by the following mechanisms, involving two steps. First, three formulations accumulated in the tumor site and reached high concentrations in the tumor, due to the enhanced permeability and retention effect (24). Secondly, it was hypothesized that TF-LP, which bound to and was internalized in tumor cells via ligand-receptor interactions, may lead to a promising accumulation in tumors compared with the other non-targeting formulations. The non-targeting formulations remained in the interstitial space and were easily identified, decomposed and phagocytosed, thereby resulting in drug release outside the cancer cells (25). In the present study, the effect of the treatment with TF-LP *in vivo* and the biodistribution of DIR-loading TF-LP in the A549-bearing nude mice indicated that TF-LP may be a novel and potent drug delivery system for targeting lung cancer and reducing the side-effects of chemotherapeutic agents to a considerable extent.

### Conclusion

The present study successfully synthesized a targeted-NP drug-delivery platform that was specific to lung cancer cells using TF and biomaterials approved by the Food and Drug Administration. The particle size, surface charge, and drug loading yield drug release rate, which are factors that may be controlled for specific therapeutic applications, were characterized. The data from the present *in vitro* DOX release experiments revealed that lipid-coated NPs undergo a sustainable, controlled release of DOX. The targeting specificity of the synthesized NPs was demonstrated, along with the enhanced cytotoxicity of the NPs against target cells and tumor spheroids compared with the non-targeted cells. In addition, the DOX-loaded TF-LP exhibited evident antitumor effects in lung cancer-bearing mice. The present platform exhibits considerable therapeutic potential due to the effective delivery of a variety of chemotherapeutic agents to lung cancer tumors in a targeted manner.

## Figures and Tables

**Table I:** Characteristics of DOX-loaded PLGA-NP, L-P and TF-LP (n=3).

Group	Particle size, nm	Polydispersity	Zeta-potential, mV	Encapsulation efficiency, %
PLGA-NP	93±8.8	0.197	−21.37±1.51	85.75±2.55
L-P	111±11.4	0.180	−22.16±1.88	94.29±1.94
TF-LP	108±12.5	0.212	−21.32±1.91	92.48±2.57

PLGA-NP, poly D,L-lactic-co-glycolic acid nanoparticles; L-P, lipid-coated PGLA-NPs; TF-LP, transferrin-modified LPs.

**Table II:** Cytotoxicity against A549 of various DOX formulations *in vitro* after 48 h incubation.

Formulations	IC_50_ in A549 cells, μg/ml
DOX	0.06200
PLGA-NP-DOX	0.00938
LP-DOX	0.00650
TF-LP-DOX	0.00330

IC_50_, concentration of DOX that resulted in cell death in 50% of cells; DOX, doxorubicin; PLGA-NP-DOX, DOX-loaded poly D,L-lactic-co-glycolic acid nanoparticles; LP-DOX, DOX-loaded lipid-coated PGLA-NPs; TF-LP-DOX, transferrin-modified DOX-loaded lipid coated NPs.
